# Influence of hydrophobic layer and delayed placement of composite on the marginal adaptation of two self-etch adhesives

**DOI:** 10.4103/0972-0707.55619

**Published:** 2009

**Authors:** Pushpa R, Suresh BS, D Arunagiri, Naveen Manuja

**Affiliations:** Department of Conservative Dentistry and Endodontics, Kothiwal Dental College and Research Centre, Moradabad, UP, India; 1 Department of Pedodontics, Kothiwal Dental College and Research Centre, Moradabad, UP, India

**Keywords:** Delayed placement, hydrophobic layer, microleakage, self-etch adhesive

## Abstract

**Aim:**

The purpose of this *in vitro* investigation was to evaluate the influence of hydrophobic layer and delay in placement of composite on marginal adaptation of two self-etch adhesive systems (XENO-III and ALL-BOND SE).

**Materials and Methods:**

Eighty class V cavities were prepared on intact, extracted human premolars and were divided into 4 groups of 10 teeth each. Group 1: Application of bonding agents as per manufacturer directions and immediate placement of composite; Group 2: Application of bonding agent and composite similar to group1, with hydrophobic layer curing before composite placement; Group 3: Application of bonding agent similar to group 1, with 2 min delay in composite placement; and Group 4: Application similar to group 2 with 2-min delay in composite placement. The specimens were restored and light cured. After thermocycling and immersion in 2% basic Fuchsin dye solution, the teeth were sectioned and dye penetration was observed under a stereomicroscope at 20× magnification. All the samples were scored and results were analyzed using Kruskal-Wallis and Mann-Whitney tests.

**Results:**

In group 1, the microleakage along the both enamel and dentin margin was significantly higher than the other groups for both the adhesive systems. There is no significant difference between groups 2, 3 and 4.

**Conclusion:**

The addition of a more hydrophobic resin layer and delay in composite placement significantly improves the marginal adaptation of self-etch adhesive resin systems.

## INTRODUCTION

Demand for esthetic restorations has placed a focus on the development of bonding systems. The purpose of using adhesives in resin composite filling techniques is to establish a durable bond between the tooth structure and filling material, which is sufficiently tight so that gap-free restoration is possible.[1] Above all, the bond has to be sufficiently strong to withstand the shrinkage forces of the resin composite during curing as these forces are directly transmitted to the bond.[2]

Today, bonding to tooth hard tissue can be accomplished by using one of two adhesion strategies: the etch-and-rinse or the self-etch approach. In contrast to the etch-and-rinse approach, the conditioning step in self-etch systems is not separated from the priming step, and therefore, demineralization and infiltration occur simultaneously.[3] The first versions of self-etch system was based on two-bottle systems with a separate etching-priming liquid followed by the application of an adhesive resin. Recently, manufacturers have further developed self-etching priming resin-based adhesives into a single solution, often referred to as “all-in-one” systems.

Although very simple in technique, studies show that these systems may not perform as well as two-step self-etching priming system and etch-and-rinse systems. This inferior performance has been attributed to certain factors. First, the etching pattern of self-etch adhesives is not as well defined as that provided by phosphoric acid.[4,5] Second, these products create very thin coatings,[6,7] which may be oxygen inhibited, resulting in a poorly polymerized adhesive layer.[8] Third, they are highly prone to phase separation as the solvent evaporates from the solution and finally they behave as permeable membranes after polymerization.[9] The later is due to the presence of water-attracting hydrophilic domains and interconnecting water-filled channels (water trees) within the polymerized adhesive permitting water to move from the underlying dentin through the adhesive.[10] Further, it has been reported that an adverse acid-base reaction and adhesive permeability may contribute to the incompatibility between some simplified adhesives to resin composite.[11,12] Hence, a clinical procedure to eliminate this kind of incompatibility should be developed.

Thus, the objectives of the study are as follows:

1. Evaluate the effect of hydrophobic layer on marginal adaptation of two one-step self-etch adhesives at enamel and dentin margin.
2. Evaluate the effect of 2-min delayed placement of composite on the marginal permeability of two one-step self-etch adhesives at enamel and dentin margin.

This study tested the null hypothesis that there is no effect of additional hydrophobic layer and delayed placement of composite on marginal permeability of one-step self-etch adhesives at enamel and dentin margin.

## MATERIALS AND METHODS

Two one-step self-etch adhesive systems XENO-III (XE, Denstply de trey, Konstanz, Germany) and ALL-BOND SE (Bisco, Inc., Schaumburg, USA) and Filtek^TM^ Z250 (3M ESPE) hybrid composite material were used.

Forty intact caries- free human premolar extracted for orthodontic purpose were selected. Any extrinsic stains or calculus deposits on teeth were cleaned using ultrasonic scaler and specimens were stored in isotonic saline until used. Class V cavity with a dimension of 3 mm occlusogingivally, 3 mm mesiodistally and 1.5 mm depth was prepared at both the buccal and lingual surfaces of each of 40 teeth, for a total of 80 cavities. The preparations were made with number 245 carbide bur in high speed under copious water coolant. After every 5 preparation, the bur was discarded and replaced with new bur. The gingival cavosurface margin of the preparation was 1.5 mm below the cementoenamel junction. The enamel margins were given 0.5 mm bevel at 45° angle by using tapered fissure bur. The specimens were randomly and equally assigned to 4 experimental groups (*n* = 10) for each of “all-in-one” adhesives, as shown in Table 1. Adhesive systems, composition and application modes of different groups were described in Table 2.

**Table 1:** Distribution of samples

Group 1	Application of bonding agent as by manufacturer and placement of composite immediately
Group 2	Application of bonding agent and composite similar to group 1, with hydrophobic layer curing before composite placement
Group 3	Application of bonding agent similar to group 1, with 2-min delay in composite placement
Group 4	Application similar to group 2, with 2-min delay in composite placement

**Table 2:** Adhesive systems: Composition and application mode of different groups

Adhesive system manufacturer	Composition	Group 1 Manufacture direction	Group 2	Group 3	Group 4
XENO III (Dentsply)	Liquid A: HEMA, ethanol, water, aerosil, stabilizer; Liquid B: pyro-ema, PEM-F, UDMA Camphorquinone stabilizers, ethyl-4 dimethyl aminobenzoate (co-intiator)	Mix liquids A and B for 5 s; Application of one thick coat of adhesive under pressure (30 s); Gentle air stream(10 s at 20 cm); Light activation (10s – 600 mW/cm^2^ Composite placement curing – 40s	Steps 1–4 from (MD) Application of one coat of liquid B Air stream to make the bond film uniform (3 s at 20 cm) Light action(10 s – 600 mW/cm^2^ Composite curing – 40 s	Steps 1–4 from (MD). Composite placement and curing after 2 min of adhesive curing	Steps 1–4 similar to group 2. Composite placement and curing after 2 min of adhesive curing.
ALL-BOND SE (Bisco)	Part I – ethanol, water, sodium benzene sulfinate; Part II –hydroxyethyl methacrylate, bis (glyceryl 1,3 dimethyacrylate) phosphate, biphenyldimethacrylate.	Mix liquids i and ii until uniform pink; Application of one thick coat of adhesive under agitation (10 s); Gentle air stream (10 s at 20cm); Light activation (10s – 600 mW/cm^2^) Composite placement curing – 40 s.	Steps 1–4 from (MD) Application of one coat of liquid B (of Xeno III) Air stream to make the bond film uniform (3 s at 20 cm) Light action (10 s – 600 mW/cm^2^ Composite curing – 40s.	Steps 1–4 from (MD) Composite placement and curing after 2 min of adhesive curing.	Steps 1–4 similar to group 2. Composite placement and curing after 2 min of adhesive curing.

MD: Manufacture direction

The restored specimens were stored in distilled water for 10 days. Then specimens were thermocycled for 1,000 cycles at 5° ± 1° and 55° ± 1° with 30 s of dwell time.

The teeth surfaces were painted with two layers of nail varnish, except for a 1 mm rim around the margin. To prevent any dye leakage through the apical foramen, it was sealed with yellow sticky wax. All the specimens were immersed in freshly prepared 2% solution of “basic fuchsin dye” for 24 h in separate containers and were labeled for identification. After drying the samples at room temperature, the teeth were sectioned buccolingually with the help of a diamond disc held in a straight hand-piece; all the samples were observed under stereomicroscope (Nikon SMZ1000) at 20× magnifications and images of sections were taken. The following ranking system was used to score degree of dye penetration.[13] 0 – No dye penetration; 1 – Dye penetration up to half of the cavity depth; 2 – Dye penetration more than the half of the cavity depth, but not extending the axial wall; and 3 – Dye penetration arriving to the cavity floor/axial wall and beyond. The data were analyzed using non-parametric Kruskal-Wallis (*P* < 0.05) and Mann-Whitney test (*P* < 0.05) in SPSS 15 software package.

## RESULTS

**Figure 1:**
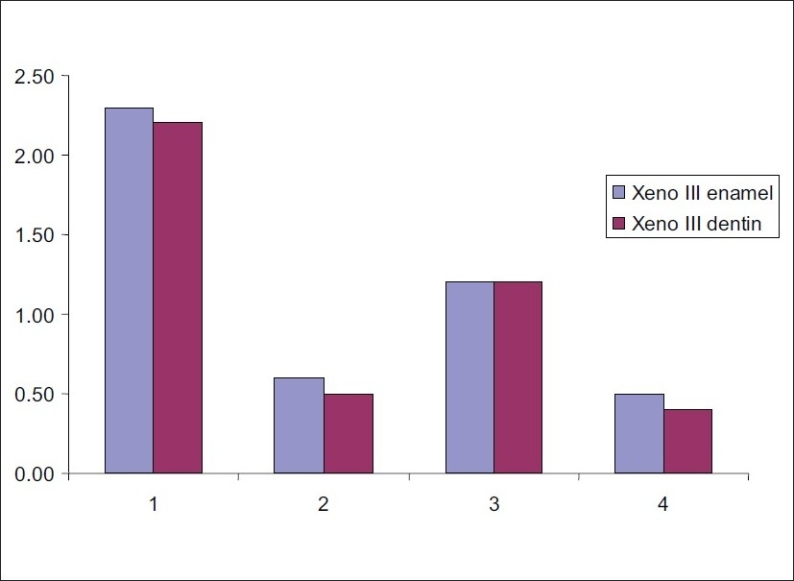
Mean scores for dye penetration at enamel and dentin margins in Xeno III of all the four groups

**Figure 2:**
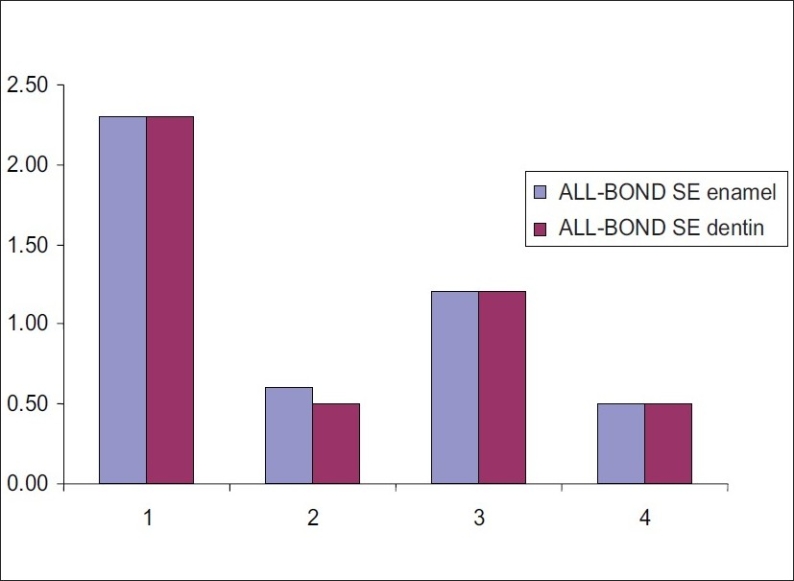
Mean scores for dye penetration at enamel and dentin margins in ALL-BOND SE of all the four groups

Tables 3 and 4 and Figures 1 and 2 respectively show the mean scores of microleakage of XENO III and ALL-BOND SE at the enamel and dentin margin. For both the adhesives, it was revealed no significant difference in microleakage between enamel and dentin in all the four groups.

**Table 3:** Mean scores for dye penetration at enamel and dentin margins in Xeno III of all the four groups

Groups	XENO III enamel	XENO III dentin
1	2.3	2.2
2	0.6	0.5
3	1.2	1.2
4	0.5	0.4

**Table 4:** Mean scores for dye penetration at enamel and dentin margins in ALL–BOND SE of all the four groups

Groups	Enamel	Dentin
1	2.3	2.3
2	0.6	0.5
3	1.2	1.3
4	0.5	0.5

When adhesive systems were used according to manufacturer's directions, i.e., group 1, there was significant increase in microleakage than the other groups for both the adhesive systems. There was no significant difference in microleakage in groups 2, 3 and 4.

## DISCUSSION

Although technological advances in materials and techniques have been developed in adhesive dentistry, shortcomings persist since long-term microleakage occurs with all restorations.[3] Perfect adhesion to tooth structure is the primary objective. However, several contributing factors, such as material physical characteristics, polymerization source, cavity location and configuration (C-factor), composition of dentin, occlusion components, lack of strict adherence to manufacturer's instructions and inconsistent clinical techniques by the practitioner, can diminish restorative success.[14]

When comparing the adhesive systems that were applied according to manufacturer's recommendation, the marginal adaptation of both the adhesives systems were found to be significantly improved in groups in which additional hydrophobic layer was applied.

In this study, liquid B of XENOIII was used as hydrophobic layer in groups 2 and 4; this was in accordance with Brackett, Tay and others.[15]

Several mechanisms could account for the better performance of these groups. First, this finding is in accordance with the study conducted by Pashley and others; they found higher *μ*TBS for Adper Prompt when a second adhesive layer was applied, followed by polymerization of the first layer. The author observed that an additional application of the bonding agent could seal the non-polymerized layer oxygen, thus enabling it to be adequately polymerized.[6] The same reason could have attributed for better performance in groups 2 and 4. Second, the additional application must have increased the concentration of the hydrophobic monomer, thereby reducing the relative concentration of solvents and hydrophilic monomers within the adhesive interface, which in turn reduced the intrinsic permeability of these one-step self-etching adhesives.[16–18] Third, this hydrophobic layer seems to limit diffusion of water through the hybrid layer to the interface between the adhesive and resin composite, otherwise this diffusion might have occurred rapidly. [19,21] Water diffused at the interface could, in turn, inhibit polymerization and thereby weaken the adhesive/resin composite interface.[22] Fourth, the additional hydrophobic layer may also have slowed the extraction of unpolymerized monomers or oligomers from the hybrid layer. Zones of poorly polymerized hydrophilic phases that permit water movement have been demonstrated within the hybrid layers and self-etching adhesives.[23] Fifth, this additional layer of resin increased the thickness of the adhesive layer, which is known to reduce polymerization stresses.[24]

The delayed placement of composite also improved the marginal adaptation of both the adhesives; this result is similar to those reported by Asaka, Miyazaki and others.[25]

In this study, two-minute delay in composite placement was selected as one of the parameters because it was found from the previous study that (Asaka, Miyazaki and others, 2006) bond strength was significantly improved in groups in which there was delay of 2 min in composite placement but there was no significant difference between the 2-min, 5-min, 10-min delays in composite placement. On the other hand, bond strength was significantly reduced in groups in which there was immediate placement or 1-min delay in composite placement.[25]

It has been suggested that etching effect of the self-etch adhesive is stopped by interaction with the mineral component of the dentin substrate followed by polymerization, which reduces the free acidic monomers.[26] From a report focusing on the effect of self-etching primers on the continuous demineralization of dentin, the etching effect of the acidic functional monomer did not stop with the polymerization of adhesives.[26] Residual acid should be consumed and neutralized by reaction with hydroxyapatite of the dentin substrate,[27] otherwise the low pH of the self-etching adhesives may persist after polymerization and will be sufficient to inhibit the polymerization of resin composite if it is placed immediately. This improper polymerization of composite at the juncture of the adhesive and the composite when it is placed immediately after light irradiation of the adhesive is thought to be caused by the presence of acidic monomers in the oxygen inhibition layers of one-step self-etch adhesives. There might be an adverse interaction between the nucleophilic tertiary amine in the composites and acidity (acidic functional monomers) of the superficial layer of the adhesives.[28–30] The delay in composite placement could have allowed for the time-dependent acid-base reaction between the remaining acidic functional monomers and the mineral component of the dentin substrate to neutralize the residual acid.[25]

The results of this study showed that the marginal adaptation of both the self-etch adhesive systems was affected by both hydrophobic layer and delay in composite placement. Therefore, the null-hypothesis has to be rejected.

## CONCLUSION

Within the limitation of this study, it can be concluded that the addition of a more hydrophobic resin layer and delay in composite placement significantly improves the marginal adaptation of self-etch adhesive resin systems.
